# Mental health in the era of COVID-19: prevalence of psychiatric disorders in a cohort of patients with type 1 and type 2 diabetes during the social distancing

**DOI:** 10.1186/s13098-020-00584-6

**Published:** 2020-08-31

**Authors:** Janine Alessi, Giovana Berger de Oliveira, Debora Wilke Franco, Bibiana Brino do Amaral, Alice Scalzilli Becker, Carolina Padilla Knijnik, Gabriel Luiz Kobe, Taíse Rosa de Carvalho, Guilherme Heiden Telo, Beatriz D. Schaan, Gabriela Heiden Telo

**Affiliations:** 1 Post-Graduate Program in Medical Science: Endocrinology, Universidade Federal do Rio Grande do Sul, Porto Alegre, Brazil; 2 Internal Medicine Department, Hospital São Lucas-Pontifícia Universidade Católica do Rio Grande do Sul, Porto Alegre, Brazil; 3 School of Medicine, Pontifícia Universidade Católica do Rio Grande do Sul, Porto Alegre, Brazil; 4 Post-Graduate Program in Medicine and Health Sciences, Pontifícia Universidade Católica do Rio Grande do Sul, Porto Alegre, Brazil; 5 School of Medicine, Universidade Federal do Rio Grande do Sul, Porto Alegre, Brazil; 6 Endocrinology division, Hospital de Clínicas de Porto Alegre, Porto Alegre, Brazil; 7 National Institute of Science and Technology for Health Technology Assessment (IATS)–CNPq, Porto Alegre, Brazil; 8 Hospital de Clínicas de Porto Alegre, HCPA, Porto Alegre, Rio Grande do Sul, Brazil; 9 Hospital São Lucas da PUCRS, Porto Alegre, Rio Grande do Sul Brazil

**Keywords:** Diabetes mellitus, Mental health, COVID-19 pandemic, Social distancing, Quarantine

## Abstract

**Background:**

In patients with diabetes, the prevalence of depression and anxiety symptoms is about two to four times greater than in the general population. The association between diabetes and mental health disorders could be exacerbated in a stressful environment, and psychological distress could increase depressive symptoms and cause adverse diabetes outcomes.

**Objectives:**

To assess the prevalence of mental health disorders in patients with diabetes during the social distancing period due to COVID-19 pandemic.

**Methods:**

This is a cross-sectional study developed to assess the impact of social distancing on a cohort of adults with type 1 (n = 52) and type 2 diabetes (n = 68) in Brazil. Inclusion criteria involved having an HbA1c test collected in the past 3 months and having a valid telephone number in electronic medical records. The primary outcome was the prevalence of minor psychiatric disorders, assessed by survey (SRQ-20). Secondary outcomes included the prevalence of diabetes related emotional distress, eating and sleeping disorders, all assessed by validated surveys at the moment of the study. Statistical analyses included unpaired *t*-test for continuous variables and *χ*^2^ test for categorical variables.

**Results:**

Overall (n = 120), participants had a mean age of 54.8 ± 14.4 years-old, and HbA1c of 9.0 ± 1.6% (75 ± 17.5 mmol/mol); 93% of patients showed signs of current mental suffering based on the surveys measured. Almost 43% of patients showed evidence of significant psychological distress, with a significant greater tendency in patients with type 2 diabetes. The presence of diabetes related emotional distress was found in 29.2% of patients; eating disorders in 75.8%; and moderate/severe sleeping disorders in 77.5%.

**Conclusions:**

We found a high prevalence of evidence of psychological distress among patients with diabetes during the COVID-19 pandemic and this highlights the need for mental health access and support for patients with type 1 and type 2 diabetes.

## Background

Diabetes mellitus and psychiatric disorders share a mutual interface: the challenge of living and overcoming diabetes may result in emotional overload, and the presence of depression and anxiety symptoms may be associated with lower treatment adherence, leading to worse glycemic control [1, 2]. In patients with diabetes, the prevalence of depression and anxiety symptoms is about two to four times greater than in the general population [3, 4]. Although more research is needed to fully understand the link between diabetes and depression, it is clear that metabolic dysregulation influences brain function and disturbances in peripheral glucose regulation might be associated with depressed mood [5, 6]. Some cases of depression might result from low levels of insulin-like growth factor (IGF) into the brain, wich was shown to produce antidepressant behavioural responses in experimental studies [6–8]. Nutrient-activated gut to brain signaling pathways also appear to play a role in the genesis of depressive symptoms. A highly significant association between leptin levels, depressed mood and sleep disturbances has been shown in normal-weight individuals [6, 9]. Also, ghrelin can exert antidepressant effects in men and carbohydrates appear to lead to ghrelin suppression [6, 10, 11].

The association between diabetes and mental health disorders could be exacerbated in a stressful environment, and psychological distress could increase depressive symptoms and cause adverse diabetes outcomes [12, 13]. The emergence of a potentially fatal pandemic represents a new reason for uncertainty and anxiety in this group of patients. Since December 2019, when a series of cases of severe pneumonia caused by a new coronavirus was described in Wuhan-China, the COVID-19 infection, as it became known, quickly spread throughout the world [14–16]. On Jul 07th, more than 11 million and 600 thousand confirmed cases have been identified worldwide, totaling 538 thousand deaths [17]. The first case of someone suffering from COVID-19 in South America was confirmed on February 26th, 2020 in São Paulo, Brazil. Since then, Brazil has recorded the largest number of cases in Latin America and recently has emerged as a new epicenter of the pandemic in the world [18].

A number of measures have been taken to prevent the spread of COVID-19, involving the isolation of suspected cases, tracking and monitoring of contacts, and dissemination of regional and national information, which included the recommendation of social distancing, especially for high risk groups such as patients with diabetes [19]. The social distancing recommendation have a psychological effect even in patients without diabetes, as shown by Talevi et al. [20]. In this review, the authors report that up to 53.8% of people experienced psychological distress during the initial stage of the COVID-19 outbreak. A range of negative psychological responses were identified, such as anxiety, depression, insomnia and worries about individuals’ own health and family. The levels of stress, anxiety and depression ranged from mild to moderate-severe [20–24]. Among patients with confirmed infection, findings show that nearly 50% of people diagnosed with COVID-19 had depressive symptoms, over 55% had anxiety and almost 70% had somatic symptoms [20, 25].

The psychological repercussion of the current scenario in patients with diabetes is still hypothetical. It is well known that those patients, due to the conditions of the underlying disease, already have a greater tendency to develop psychiatric disorders throughout life. It is possible that the COVID-19 pandemic, as well as the social isolation determined by it, may interfere with parameters of mental health in patients with diabetes. The present study aimed to investigate the impact of the current pandemic on the prevalence of mental health disorders in patients with type 1 and type 2 diabetes.

## Methods

### Study design and setting

This is a cross-sectional study developed to assess the prevalence of mental health disorders in a cohort of patients living with diabetes during the social distancing period due to COVID-19 pandemic. Electronic medical records were used to select patients with diabetes in a follow-up at the Endocrinology department of a public hospital in Southern Brazil. Patients who met the inclusion criteria received a telephone call for an invitation and application of the informed consent form (by electronic means or audio recording). Participants who agreed to participate in this study received a second phone call for data capture. All the study procedures started 1 month after the disclosure of the national ordinance that standardizes the social distancing recommendation for risk groups, including diabetes, in Brazil. At the time of the evaluation, the state of Rio Grande do Sul followed the *Contingency Plan and State Action for the Prevention of the Human Infection COVID*-*19*, which restricted the functioning of establishments that offer essential services and which regulated the indication of an exceptional teleworking regime for people with respiratory diseases, immunosuppressed or with chronic disease, upon medical recommendation. All data were collected in 8 days in order to have the same pandemic time for all participants. All contacts were made by telephone by trained researchers in order to preserve participants from social exposure. All data collected during the phone calls were recorded directly on an electronic database validated by the study staff.

### Participants

Patients with type 1 and type 2 diabetes in a regular follow-up at the Endocrinology outpatient clinic, who attended a medical appointment in a one-year period, for type 2 diabetes, and three-year period for type 1 diabetes (from 2016 to 2019), were identified in an electronic database. Inclusion criteria involved age ≥ 18 years old, an hemoglobin A1c (HbA1c) test collected between January and March 2020 at the hospital laboratory, and having a valid telephone number in the electronic medical record. Patients who had any physical or cognitive impairment that prevented the application of the study questionnaires (such as dementia and severe hearing impairment), as well as patients who were hospitalized at the time of the study, were excluded.

### Variables and data sources

The primary outcome assessed was the prevalence of minor psychiatric disorders among patients with type 1 and type 2 diabetes. Secondary outcomes included the prevalence of diabetes-related emotional distress, eating disorders and sleeping disorders at the moment of the study.

For the assessment of psychological distress, such as anxiety and depression, the Brazilian validated version of the *Self Report Questionnaire*-20 (SRQ 20) was used [26, 27]. This 20-item questionnaire addresses questions related to physical and psychoemotional symptoms that may have been presented in the past 30 days, asking yes or no questions. A positive screening for minor psychiatric disorders was considered when the survey scored greater than or equal to 7, which was considered a sign of current mental suffering.

Diabetes-related emotional distress was assessed by the Brazilian validated version of the Problem Areas in Diabetes Scale (B-PAID), which is a 20-item questionnaire that contemplates the patient’s perspective on the impact of certain issues related to diabetes on a 4-point response scale, with responses ranging from 0 = “it is not a problem” to 4 = “it is a serious problem”. The scores for each item were summed up, and then multiplied by 1.25 to generate a total score out of 100. Severe diabetes emotional distress was considered present when the score was greater than or equal to 40 [28, 29].

The prevalence of eating disorders was assessed by the Brazilian validated version of the Eating Attitudes Test (EAT-26). This survey addresses 26 issues related to eating habits and attitudes on a 3-point response scale, with responses ranging from 0 = “never” to 3 = “always”. The presence of a significant eating disorder was considered when the score was greater than or equal to 20 [30, 31].

To assess sleep disorders, the Brazilian version of the Mini Sleep Questionnaire (MSQ), a 10-item scale, was used on a 7-point response scale, with responses ranging from 1 = “never” to 7 = “always”. A sleep disorder (moderate or severe) was considered when a score greater than or equal to 28 was present [32, 33].

It should be noted that the scales used to assess mental health disorders were designed for self-application. The fact that those scales were applied by telephone contact could be a potential source of bias. To minimize this effect, the researchers strictly followed the steps of the questionnaires, repeating the alternative answers to each question only when requested to be as accurate as possible.

Demographics and clinical data, such as the presence of comorbidities, continuous use medications, weight and height—obtained from the last visit for calculating the body mass index (BMI) –, and HbA1c (high-performance liquid chromatography method) data from the last 3 months were collected from electronic medical records. Cardiovascular disease was considered present if there was a previous history of coronary heart disease, stroke, or heart failure registered in medical records. The presence of diabetes complications was also documented according to medical records. The presence of retinopathy was considered based on the last registered fundus examination. For neuropathy, it was considered the presence of a documented diagnosis of previous neuropathy or a monofilament 10 g test altered in the last medical appointment. For diabetic nephropathy, it was considered the presence of microalbuminuria or chronic kidney disease in which the etiology was attributed to diabetes in the medical records.

Some clinical data, such as the use of antidepressant or anxiolytic drugs and previous diagnosis of psychiatric disorders, were obtained from both electronic medical records and checked directly with the patients during the phone calls. Previous diagnosis of common mental disorder was considered if there was a previous or current diagnosis of depressive episode, major depressive disorder or anxiety disorders. Compliance with the recommendation of social distance was questioned directly to the participants according to the follow: (1) total social distancing was considered when the patient did not leave the house under any circumstances; (2) partial social distancing was considered when the patient left the house only for basic activities (such as going to the market and pharmacy); or (3) no social distancing, when the patient maintained regular activities.

The institutional ethics committee approved the study protocol (Number 4.029.368), and all authors signed the confidentiality document for data use.

### Sample size

The sample size was calculated for a prevalence survey with finite population correction. Considering that the prevalence of anxiety and depression disorders among patients with type 1 was 17.6% and type 2 diabetes was 16%, we considered a mean prevalence known of 17%. The calculation was performed taking into account that in 2019 there were 16.8 million individuals with diabetes in Brazil. The number required for an analysis with 5% accuracy and 85% confidence level was 117 patients [4, 34, 35]. The number of patients included with type 1 diabetes and type 2 diabetes was determined randomly. The limitations imposed by the current pandemic and the recommendation of social distancing to patients with diabetes added difficulty in approaching and contacting a greater number of patients, which motivated the choice of the 85% confidence level.

### Statistical methods

Analyses were performed using SPSS Statistics 20. Descriptive data are presented as mean and standard deviation (SD) or percentages. The data distribution was analysed and, since it had a normal distribution, parametric tests were used. In order to evaluate possible differences according to diabetes type, statistical analyses included unpaired *t*-test for continuous variables and *χ*^2^ test for categorical variables. The primary outcome (minor psychiatric disorders) was then evaluated as the dependent variable in a multivariable logistic regression model designed to control for possible confounders in the interaction between the primary outcome and the diabetes type. An α level of ≤ 0.05 was used to determine statistical significance. This study followed the STROBE statement for the reporting.

## Results

### Participant characteristics

A total of 245 potentially eligible patients were identified, and 146 were randomly recruited to participate in the study. The recruitment stopped after inclusion of the planned sample size, when 120 individuals, 52 with type 1 and 68 with type 2 diabetes, agreed to participate and provided informed consent (see Additional file 1: Figure S1). Age, sex, diabetes duration, and HbA1c levels did not differ by enrollment status (data not shown).

Overall (n = 120), participants had a mean age of 54.8 ± 14.4 years old; 55.8% were female, 85.8% white and 76.7% overweight/obese. The mean diabetes duration was 21.8 ± 10.9 years and the HbA1c value was 9.0 ± 1.6% (75 ± 17.5 mmol/mol) (see Table 1). Patients with type 2 diabetes were older (62.3 ± 9.1 vs. 45.0 ± 14.2 years of age; *p* < 0.001), had a greater racial representation (22.1% vs. 3.8% not white; *p* = 0.02) and a higher prevalence of overweight and obesity (95.6% vs. 51.9%; *p *< 0.001), when compared with patients with type 1 diabetes. Although younger, type 1 diabetes patients had a longer diabetes duration (25.2 ± 11.5 vs. 19.2 ± 9.7 years; *p* < 0.01). Both groups were comparable with respect to HbA1c levels and presence of diabetes complications.

**Table 1:** Demographics and clinical characteristics of study participants

	Total (n = 120)	Type 1 diabetes (n = 53)	Type 2 diabetes (n = 68)	*P* value
Age (years)	54.8 ± 14.4	45.0 ± 14.2	62.3 ± 9.1	< 0.001
Sex (% female)	55.8%	48.1%	61.8%	0.13
Race/ethnicity (% white)	85.8%	96.2%	77.9%	0.02
Diabetes duration (years)	21.8 ± 10.9	25.2 ± 11.5	19.2 ± 9.7	< 0.01
Age at diabetes diagnosis (years)	32.7 ± 16.1	19.8 ± 12.7	42.8 ± 10.3	< 0.001
HbA1c (%) (mmol/mol)	9.0 ± 1.6 75 ± 17.5	8.8 ± 1.5 73 ± 16.4	9.1 ± 1.7 76 ± 18.6	0.29
Diabetes complications				
Retinopathy	48.3%	55.8%	42.6%	0.15
Neuropathy	30.0%	30.8%	29.4%	0.87
Nephropathy	40.0%	38.5%	41.2%	0.76
Insulin use (%)	92.5%	100%	86.8%	< 0.01
Metformin use (%)	42.5%	0%	75%	< 0.001
BMI (% overweight/obese)	76.7%	51.9%	95.6%	< 0.001
Systemic arterial hypertension (%)	58.3%	30.8%	79.4%	< 0.001
Cardiovascular disease	29.2%	15.4%	39.7%	< 0.01
ACE inhibitors use	46.7%	30.8%	58.8%	< 0.01
Previous diagnosis of common mental disorders^a^	23.3%	25.0%	22.1%	0.70
Social distancing (% total/partial^b^)	92,5%	88,5%	95,6%	0.14

Data are mean ± standard deviation or %. An α level of ≤ 0.05 indicates significant difference. BMI, Body mass index; HbA1c, hemoglobin A1c. ACE, Angiotensin-converting enzyme; ^a^ Common mental disorders, which includes depressive episode, major depressive disorder and anxiety disorders. ^b^ Social distancing includes patients who followed the orientation of total (home-staying only) or partial social isolation (left home only for basic activities, such as market, pharmacy and health care)

From the whole group, 9 patients (7 type 1, and 3 type 2 diabetes) had some previous serious psychiatric diagnosis, which were not considered as common mental disorders in analysis. Among patients with type 1 diabetes, 3 had a diagnosis of schizophrenia; 1 bipolar mood disorder; 1 borderline personality disorder; and 1 self-mutilation history. Among patients with type 2 diabetes, 1 patient had a previous diagnosis of schizophrenia and 1 had a diagnosis of obsessive–compulsive disorder. There was no difference between type 1 and type 2 diabetes regarding the presence of previous common mental disorders. Regarding social distancing, in total, 42.5% of patients were following the guidance of total isolation, 50% were on partial social distancing (leaving home only for basic activities) and only 7.5% were not on doing any type of social distancing (keeping regular daily activities). There was no difference between groups with regard to social distancing.

### Survey results

In the studied participants, 93.3% (94.2% in type 1 and 92.6% in type 2 diabetes, *p* = 0.73) had some sign of a psychiatric disorder, which was assessed by a positive screening in at least one of all the specific scales measured in this study (minor psychiatric disorders, diabetes-related emotional distress, and eating and sleeping disorders).

**Fig. 1:**
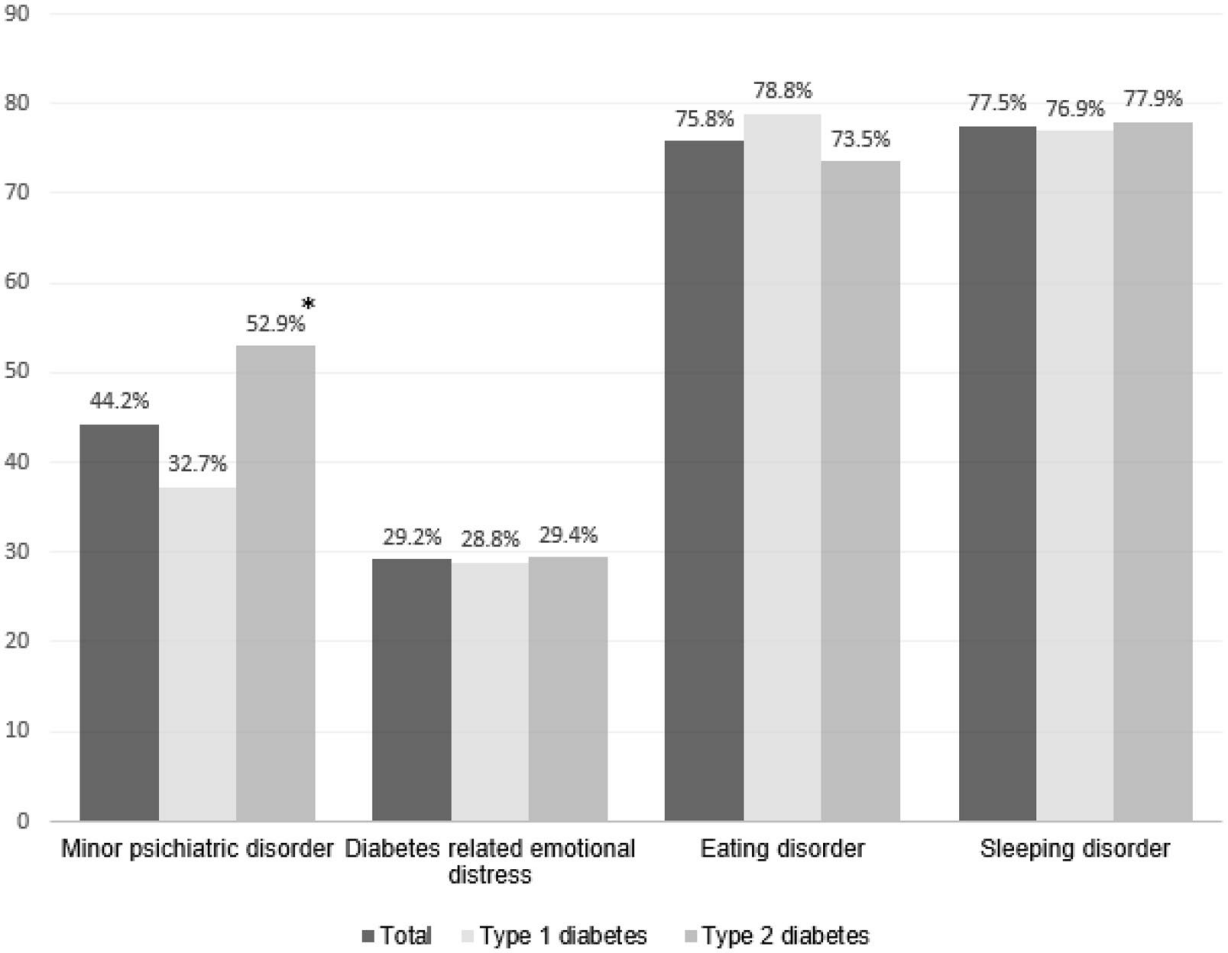
Prevalence of positive screening for psychiatric disorders among patients with type 1 and type 2 diabetes. The *Self Report Questionnaire*-20 (SRQ 20) was used for the assessment of minor psychiatric disorders, such as anxiety and depression. Diabetes related emotional distress was assessed by the Brazilian Problem Areas in Diabetes Scale (B-PAID). The prevalence of eating disorders was assessed by the Eating Attitudes Test (EAT–26). The Mini Sleep Questionnaire (MSQ) was used to assess sleep disorders. *P = 0.03

Regarding the primary outcome, the presence of psychological distress, that measure depressive and anxiety symptoms, 44.2% of patients had a positive screening based on the SRQ 20 (see Fig. 1). In the type 1 diabetes group, this prevalence was 32.7%, while in the group with type 2 diabetes, the prevalence of psychological distress was higher, 52.9% (*p *= 0.03). The question number 17 of the SRQ 20 (“*has the thought of ending your life been on your mind*“) addresses suicidal ideation, and, overall, 6.7% of patients had a positive response to this item. Also, considering all the demographic and clinical differences between type 1 and type 2 diabetes patients showed in Table 1, we performed a multivariable logistic regression to evaluate the impact of variables of clinical interest on the interaction between the primary outcome (minor psychiatric disorders) and the type of diabetes (see Table 2). We included into the model; age, sex, race/ethnicity, age of diabetes diagnosis, HbA1c, BMI, previous common mental disorders, and social distancing. The adjusted Odds Ratio and its 95% confidence interval for the interaction between minor psychiatric disorders and type 2 diabetes was 7.60 (1.97–29.34).

**Table 2:** Multivariable logistic regression to identify predictors of minor psychiatric disorders

	Odds ratio	Confidence interval (95%)	*P* value
Age at diagnosis (per 1 year increase)	0.96	0.92–0.99	0.04
Sex (female)	2.24	0.95–5.32	0.06
BMI (eutrophic)	1.77	0.56–5.56	0.33
Age (per 1 year increase)	1.01	0.97–1.06	0.66
Previous diagnosis of common mental disorders	1.15	0.43–3.11	0.77
HbA1c (per 1% increase)	1.03	0.79–1.35	0.81
Race/ethnicity (white)	1.01	0.28–3.64	0.98
Social distancing	2.04	0.35–11.81	0.42
Type 2 diabetes	7.60	1.93–29.71	0.004

Multivariable logistic regression model to assess predictors of the presence of minor psychiatric disorders (*χ*^2^ = 17.94, *p* 0.05, R^2^ Negelkerke 0.19). BMI, Body mass index; HbA1c, hemoglobin A1c. Common mental disorders includes depressive episode, major depressive disorder and anxiety disorders. Social distancing includes patients who followed the orientation of total or partial social detachment (left home only for basic activities, such as market, pharmacy and health care)

Secondary outcomes included the prevalence of diabetes-related emotional distress, eating disorders and sleeping disorders. The presence of diabetes-related emotional distress was found in 29.2% of patients; eating disorders in 75.8%; and moderate/severe sleeping disorders in 77.5% of patients (Fig. 1). There was no significant difference in these outcomes between patients with type 1 and type 2 diabetes. In the type 1 diabetes group, the prevalence of diabetes-related emotional distress was 28.8% vs. 29.4% in the type 2 diabetes group (*p *= 0.95). For the eating disorders evaluation, 78.8% of patients with type 1 diabetes showed a positive screening for eating disorders *vs*. 73.5% of those living with type 2 diabetes (*p* = 0.50). In the analysis of sleep pattern, 76.9% of patients with type 1 diabetes showed signs of moderate/severe sleep disorder vs. 77.9% of those living with type 2 diabetes (*p* = 0.89). We also performed a multivariable logistic regression to evaluate the impact of BMI on the interaction between the positive screening for eating and sleeping disorders and the type of diabetes, and no significant interaction was identified (data not shown).

## Discussion and conclusions

In this study, we sought to investigate psychological characteristics of people living with diabetes after 1 month of social distancing recommendations in Brazil. We found a high prevalence of significant psychological distress among patients with type 1 and type 2 diabetes, with approximately 93% of the studied patients showing signs of current mental suffering in some psychological specific area. Almost half of the patients had a positive screening for psychological distress, such as anxiety and depression, with a significant greater tendency in patients with type 2 diabetes. The presence of diabetes-related emotional distress was present in only 29.2% of the interviewees, which does not appear to directly justify the high prevalence of psychiatric disorders found in this study. Approximately three out of four patients had a positive screening for eating and sleeping disorders, which may reflect the systemic repercussion of a latent anxiety condition.

It is well documented that depression and anxiety are more prevalent among patients with diabetes when compared to general population [36–38]. An epidemiological study by Meurs et al. evaluating more than 90,000 patients found an 80% increased risk of depression and anxiety in patients with diabetes [36]. The data described in the literature shows a co-prevalence of diabetes and depression ranging from 17.6 to 21% [39, 40]. In Brazil, the prevalence of depression in patients with diabetes, in usual situations, appears to be similar to that found in other countries, reaching 22% in the most recently published study [41]. Considering the current scenario, a study by Huang et al. in China showed a prevalence of anxiety and depression in the general population of 35% and 20%, respectively [42]. This makes us reflect about the possible impact that 1 month of social distancing, associated with all the stressors related to the current pandemic, has on this group of patients. Health appointments not fully available, difficulties in obtaining diabetes medications and supplies, besides the lack of scientific information regarding the real relationship between COVID-19 and diabetes, may have contributed to the high prevalence of psychological distress found in this study. The possible vulnerability intrinsic to diabetes seems to be exacerbated in the current scenario.

It is important to notice that the COVID-19 pandemic may impact patients with type 1 and type 2 diabetes differently. There is a tendency for a higher prevalence of depression in patients with type 2 diabetes when compared to those with type 1 diabetes in normal situations. A study performed by Bak et al. showed that patients with type 2 diabetes had almost twice the prevalence of depression symptoms when compared to those with type 1 diabetes [43]. In addition, intrinsic differences in types of diabetes can be affected in different ways during the period of social distancing. In type 1 diabetes, which requires precision in terms of the amount and timing of insulin administration, having more time at home could result in improved adherence and disease control. On the other hand, in type 2 diabetes, the maintenance of healthy habits, including physical exercise and balanced diet, can be greatly impaired during quarantine. These possible differences can have a positive or negative impact in terms of glycemic control, contributing differently to the appearance of psychological distress during the COVID-19 pandemic. It should be noted that these differences are still hypothetical, requiring specific studies for a better understanding.

Besides the high prevalence of psychological distress, our findings highlight the observation that the risk of suicide may be increased during the period of social distancing by COVID-19 in patients with diabetes. In the studied cohort, almost 7% of patients expressed positive responses to the question “*has the thought of ending your life been on your mind*?” in the SRQ 20. It is important to notice that the questionnaires were applied in a single phone call interview generated by researchers who had no bond or previous connection with participants. It is possible that, if applied under other conditions, this number would be even higher. Our findings are compatible with what was exposed by Gunnel et al., which stated that the pandemic would cause distress and leave many people vulnerable to mental health problems and suicidal behavior. Mental health consequences are likely to be present for longer and peak later than the actual pandemic [44]. This reinforces the importance of the active and ongoing participation of mental health professionals in policy task forces during this critical period [45].

Our study also showed a high prevalence of eating disorders among patients with diabetes after 1 month of social distancing. Literature data show that approximately 14% to 35% of patients with diabetes have a positive screening for eating disorders when assessed by EAT-26, a percentage much lower than the one found in our cohort [46, 47]. A pilot study by Fernandez-Aranda et al. demonstrated that, after just 2 weeks of confinement, almost 38% of patients reported symptoms related to eating disorders. The authors reflect that concerns about health and fitness during confinement might serve as a precipitating factor for the development of an eating disorder in vulnerable individuals [48]. It is important to note that our study was carried out after a longer period of social distancing, but in milder confinement conditions, different from the lockdown measures evaluated in the study by Fernandez-Aranda et al. Nevertheless, although not evaluated in our study, the high prevalence of eating disorders in this population could interfere in diet and, consequently, in glycemic control.

Another relevant aspect of our study was the high prevalence of sleep disorders in patients with diabetes during this period. Only one study was carried out to assess sleep quality during the COVID-19 pandemic, which showed 18% prevalence of sleeping disorders [43]. We believe that the presence of a positive screening for moderate and severe sleep disorder in our cohort is possibly multifactorial: the presence of obstructive sleep apnea in the groups with highest BMI, eventual nocturnal hypoglycemia episodes, staying longer time at home, practicing less physical activity, and having irregular sleep times may play an important role in this variable. In addition, it is possible that the presence of insomnia in this period reflects an anxiety sign related to a heightened concern about the risk of having COVID-19 while having diabetes. These hypotheses are merely speculation, requiring specific studies for better understanding.

It is important to highlight some limitations of the present study. This study involved a cross-sectional research design and data regarding mental health that was not assessed before the period of social distancing for comparison in this same population. The absence of a control group without diabetes is also a limitation of the study. It must be taken into account that a relatively small sample was included in this study, although in accordance to the sample size calculation. In addition, patients were selected from a single tertiary center, which can limit external validity. Some parameters, such as labor activities and patients’ current purchasing power, and specific information on the use of antipsychotics and mood stabilizers were not available in the electronic medical records and were not assessed directly with the participants, not allowing us to interpret the medication use and economic impact of this period in mental health.

Some limitations should be considered in relation to the scales used. The scales used to assess psychiatric disorders work as screening tools and have no diagnostic value. The scales used were originally validated for self-application and, in our study, they were applied by researchers through phone calls due to the limitations imposed by the current scenario. The self-report-questionnaire (SRQ-20), which was used to assess the primary outcome, has validation for minor psychiatric disorders screening in primary health care in Brazil [48]. Despite not presenting validation for screening in patients in tertiary care, patients with diabetes mellitus are included in primary care samples. For the assessment of eating disorders, the eating attitudes test (EAT-26) was used. Although there are no validation studies in the population with diabetes, their results are widely generalizable and used for screening, indicating food preoccupation and restriction [30, 31]. The EAT-26 is often indicated as one of the methods of choice for the initial assessment of eating disorders in patients with diabetes, according to Young-Hyman et al. [49]. The mini sleep questionaire (MSQ) used to assess sleep disorders has validation only for the general population and there are no specific studies on its use in patients with diabetes [32, 33].

Despite not having a diagnostic purpose, this study found a high number of patients showing evidence of significant psychological distress among patients with type 1 and type 2 diabetes during the COVID-19 pandemic. Our results serve as an alarm for the impact that the current scenario may have on the mental health of patients with diabetes. The data from this study highlight the need for mental health access and support for patients with type 1 and type 2 diabetes during and after this pandemic. Future studies and actions should address the impact of strategies to care for mental health in diabetes and to prevent glycemic control deterioration during a quarantine period.

## Supplementary information

**Additional file 1: Figure S1.** Number of patients with type 1 and type 2 diabetes who were screened, recruited, and included in the study.

## Data Availability

The data collected for the study, including deidentified participant data and informed consent form, will be available for 1 year after publication of the article upon justified request to the e-mail address of the main researcher and with a signed data access agreement.
