# Supplementary material for: Mental health in the era of COVID-19: prevalence of psychiatric disorders in a cohort of patients with type 1 and type 2 diabetes during the social distancing
Source: Diabetol Metab Syndr. 2020 Aug 31;12:76. doi: 10.1186/s13098-020-00584-6 (PMC7457442; doi:10.1186/s13098-020-00584-6)

Supplementary figure 1. Number of patients with type 1 and type 2 diabetes who were screened, recruited, and included in the study.


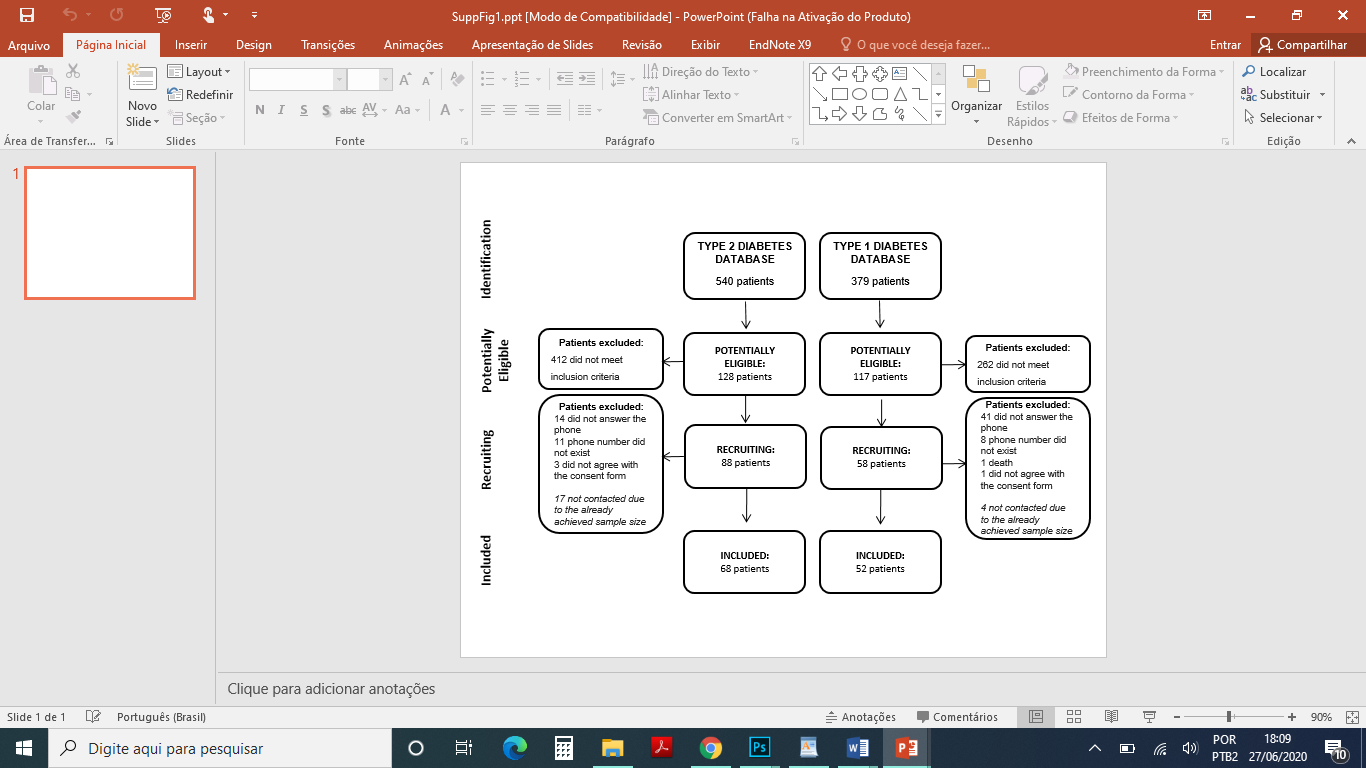

Supplement: Supplementary file 1 — Additional file 1: Figure S1. Number of patients with type 1 and type 2 diabetes who were screened, recruited, and included in the study. [file 13098_2020_584_MOESM1_ESM.docx]
